# Aspirin eugenol ester alleviates lipopolysaccharide-induced acute lung injury in rats while stabilizing serum metabolites levels

**DOI:** 10.3389/fimmu.2022.939106

**Published:** 2022-07-29

**Authors:** Qi Tao, Zhen-Dong Zhang, Zhe Qin, Xi-Wang Liu, Shi-Hong Li, Li-Xia Bai, Wen-Bo Ge, Jian-Yong Li, Ya-Jun Yang

**Affiliations:** Key Lab of New Animal Drug Project of Gansu Province, Key Lab of Veterinary Pharmaceutical Development of Ministry of Agriculture and Rural Affairs, Lanzhou Institute of Husbandry and Pharmaceutical Sciences of CAAS, Lanzhou, China

**Keywords:** aspirin eugenol ester (AEE), acute lung injury, inflammation, oxidative stress, metabolites

## Abstract

Aspirin eugenol ester (AEE) was a novel drug compound with aspirin and eugenol esterified. AEE had various pharmacological activities, such as anti-inflammatory, antipyretic, analgesic, anti-oxidative stress and so on. In this study, it was aimed to investigate the effect of AEE on the acute lung injury (ALI) induced by lipopolysaccharide (LPS) in rats. *In vitro* experiments evaluated the protective effect of AEE on the LPS-induced A549 cells. The tumor necrosis factor-α (TNF-α), interleukin-6 (IL-6), and interleukin-1β (IL-1β) were measured in the cell supernatant. The Wistar rats were randomly divided into five groups (*n *= 8): control group, model group (LPS group), LPS + AEE group (AEE, 54 mg·kg^−1^), LPS + AEE group (AEE, 108 mg·kg^−1^), LPS + AEE group (AEE, 216 mg·kg^−1^). The lung wet-to-dry weight (W/D) ratio and immune organ index were calculated. WBCs were counted in bronchoalveolar lavage fluid (BALF) and total protein concentration was measured. Hematoxylin-Eosin (HE) staining of lung tissue was performed. Glutathione (GSH), glutathione peroxidase (GPx), catalase (CAT), antioxidant superoxide dismutase (SOD), total antioxidant capacity (T-AOC), lactate dehydrogenase (LDH), C-reactive protein (CRP), myeloperoxidase (MPO), malondialdehyde (MDA), macrophage mobility inhibitory factor (MIF), TNF-α, IL-6, and IL-1β activity were measured. The metabolomic analysis of rat serum was performed by UPLC-QTOF-MS/MS. From the results, compared with LPS group, AEE improved histopathological changes, reduced MDA, CRP, MPO, MDA, and MIF production, decreased WBC count and total protein content in BALF, pro-inflammatory cytokine levels, immune organ index and lung wet-dry weight (W/D), increased antioxidant enzyme activity, in a dose-dependent manner. The results of serum metabolomic analysis showed that the LPS-induced ALI caused metabolic disorders and oxidative stress in rats, while AEE could ameliorate it to some extent. Therefore, AEE could alleviate LPS-induced ALI in rats by regulating abnormal inflammatory responses, slowing down oxidative stress, and modulating energy metabolism.

## 1 Introduction

Acute lung injury (ALI) is a type of acute inflammatory disease that is prone to develop into acute respiratory distress syndrome (ARDS), which has a high morbidity and mortality (1–3). The development of ALI can be caused by many factors, such as pneumonia, shock, trauma, burns, severe septic infection, and other non-cardiac diseases (4, 5). Until now, there is still a lack of effective drugs in the treatment of ALI (6, 7). ALI is characterized by pulmonary edema, production of pro-inflammatory mediators, uncontrolled oxidative stress, and inflammatory response, finally leading to impaired lung function (8, 9). The potential pathogenesis of ALI is the imbalance between the inflammatory and anti-inflammatory responses of the body, which leads to the development of various diseases (10). Studies suggest that there may be some preventive effect on the development of ALI by modulating the relationship between inflammation and anti-inflammation (11).

LPS is one of the important components of the cell wall of gram-negative bacteria, which consists of lipids and polysaccharides (12, 13). LPS can induce inflammatory cell infiltration and inflammatory factor release in the organism, causing strong immune responses in the body (14, 15). Therefore, LPS has been widely used to simulate inflammatory responses, including ALI (16, 17). Studies have shown that LPS could induce lung injury through several inflammatory mechanisms. When animal organisms were injected with LPS, the immune system was activated and released large amounts of pro-inflammatory cytokines, which disrupted the inflammatory and anti-inflammatory balance, leading to the development of lung inflammation (18, 19).

AEE, which is a novel medicinal compound, is synthesized by esterification of aspirin with eugenol using the prodrug principle. AEE had pharmacological activities against inflammatory (20), oxidative stress (21–24), hypolipidemia (25, 26), atherosclerosis (27, 28), thrombosis (29, 30), and anti-acute liver injury (31). In previous studies, aspirin had been found to effectively inhibit paraquat-induced oxidative stress injury, inflammatory response, and pulmonary edema, thereby alleviating paraquat-induced ALI (32). Aspirin pretreatment decreased NF-κB expression, showing a good protective effect against hyperoxia-induced ALI (33). Aspirin provides excellent protection against severe bleeding-induced ALI (34, 35). Therefore, it is speculated that AEE may also have some preventive and palliative value in ALI.

In this study, the protective effect of AEE against LPS-induced inflammation in A549 cells was firstly investigated, and then AEE was investigated against LPS-induced ALI among rats. It was aimed to explore the possibility of AEE as a novel drug for the treatment of ALI patients and promote the comprehensive utilization of AEE.

## 2 Materials and methods

### 2.1 Reagents and kits

Human alveolar epithelial A549 cells was obtained from American Type Culture Collection (ATCC, Manassas, VA, USA). AEE (purity 99.5%) was prepared in Lanzhou Institute of Husbandry and Pharmaceutical Sciences of CAAS (Lanzhou, China). LPS from *Escherichia coli* 055: B5 was obtained from Solarbio (Beijing, China). Ham’s F-12K medium, 0.05% Trypsin-EDTA, and fetal bovine serum were from Gibco (Grand Island, NY, USA). The enzyme-linked immunosorbent assay (ELISA) kits for determinations of IL-6, IL-1β, TNF-α, GSH, GPx, CAT, SOD, T-AOC, LDH, CRP, MPO, MDA and MIF were produced by Shanghai Mlbio (Shanghai, China). Cell counting kit-8 was purchased from Beyotime (Shanghai, China). BCA protein assay kit was supplied by Solarbio (Beijing, China). Cell culture plate was purchased from Corning (Beijing, China). Carboxymethylcellulose sodium (CMC-Na) was supplied by Tianjin Chemical Reagent Company (Tianjin, China). The other reagents with analytical grade were purchased from Sinopharm group (Shanghai, China).

### 2.2 Cell culture and treatments

A549 cells were cultured in 10% fetal bovine serum (FBS), 90% Ham’s F-12K medium media at 37°C under humidified atmospheric conditions containing 5% CO_2_. When reaching 90% ~ 95% confluency by observation under an inverted microscope, they were subcultured with 0.05% trypsin-EDTA. Then the groups were randomly divided into three groups (control, LPS and AEE pretreatment group).

### 2.3 Cell viability assays

To test the viability effects of AEE and LPS on A549 cells, the cell counting kit-8 (CCK-8) assay was performed. Briefly, 1×10^5^ cells were seeded in 96-well plates. After treatment, 10 µL CCK-8 reagent was added to each well. Finally, the number of viable cells were assessed by measurement of the absorbance at 450 nm (Multiskan™ FC; Thermo Scientific™, USA).

### 2.4 LPS stimulation in A549 cells

A549 cells (1 × 10^5^ cells) were seeded in 6-well plates, and incubated in a humidified atmosphere at 37°C with 5% CO_2_ for 24 h until fully attached. The cells were treated with different concentrations of AEE (4, 8 and 32 μM) for 24 h, which were selected according to the cytotoxicity, and then stimulated with LPS (10 μg·mL^-1^) for 24 h. The cell-free supernatants were collected, and stored at -80°C for cytokine assays.

### 2.5 Drug pretreatment

First, 0.5% CMC-Na solution was made up. AEE was dissolved in 0.5% CMC-Na solution to form the storage suspension solution of 40 mg·mL^-1^. LPS was dissolved in saline to form the storage solution of 5 mg·mL^-1^. The amount of drug administered to the rats were calculated based on the recorded body weight of the rats as AEE (54, 108 and 216 mg·kg^-1^ body weight) and LPS (5 mg·kg^-1^ body weight).

### 2.6 Animal experiment

Sixty male specific pathogen-free Wistar rats (7 weeks old) weighing 160 ~ 180 g were bought from the Lanzhou Veterinary Research Institute, Chinese Academy of Agricultural Sciences (Lanzhou, China). All animals were housed in groups in SPF-rated laboratory with controlled relative humidity (55-65%), 12-hour light/dark cycle and temperature (24 ± 2°C), and food and water were offered *ad libitum*. The rats were randomly divided into five groups (*n* =  8), such as control, model (LPS group), LPS + AEE low (AEE, 54 mg·kg^-1^), LPS + AEE middle (AEE, 108 mg·kg^-1^) and LPS + AEE group high (AEE, 216 mg·kg^-1^). *In vivo* models of ALI in Wistar rats were established using intraperitoneal injection (*i.p.*) of LPS (5 mg·kg^-1^). Briefly, Wistar rats were given either the vehicle alone (0.5% CMC-Na) or the vehicle combination containing AEE (54, 108 and 216 mg·kg^-1^·d^-1^) for 14 d. On the 14^th^ day of the experiment, all rats were fasted for 12 h and then dosed with 0.5% CMC-Na and different doses of AEE by gavage. After 60 min, LPS (5 mg·kg^-1^) were injected as *i.p*. into each group, except for the control group. After 6 h of LPS treatment, animals were anesthetized as *i.p.* with 80 mg·kg^-1^ sodium pentobarbital. The blood samples were collected by cardiac puncture for further analysis. Then the lung tissue was carefully harvested, quick-freezed in liquid nitrogen, and then stored at -80°C. All experimental protocols and procedures were approved by the Institutional Animal Care and Use Committee of Lanzhou Institute of Husbandry and Pharmaceutical Science of Chinese Academy of Agricultural Sciences (Approval No. NKMYD20201123; Approval Date: 23 November 2020). Animal welfare and experimental procedures were performed strictly in accordance with the Guidelines for the Care and Use of Laboratory Animals issued by the US National Institutes of Health.

### 2.7 Collection and analysis of bronchoalveolar lavage fluid

After blood samples were collected by cardiac puncture, their chest cavities were opened, and then the cannula was inserted into the trachea and the lung lobes were flushed three times, each time with 1 mL saline. The bronchoalveolar lavage fluid (BALF) were centrifuged at 2500 rpm for 10 min at 4°C, and the supernatants were collected and stored at -80°C for subsequent testing. The BALF protein concentrations were analyzed by the bicinchoninic acid (BCA) method using the BCA kit (Solarbio, Shanghai, China) according to the manufacturer’s instructions. The absorbance was measured at 562 nm and the protein content was calculated. The BALF was performed the white blood cell count.

### 2.8 Measurement of wet-to-dry ratio of the lungs

The wet/dry weight ratio of lung tissue was measured. Briefly, wet weight was determined by excision of the left lower lung lobe, and dry weight was determined through putting the lung lobe in an oven at 65°C for 72 h to remove all moisture, and then weighed the dried lung.

### 2.9 Measurement of immune organ index

The spleen tissues were collected to calculate the immune organ index. Immune organ index = spleen index (mg)/rat body weight (g).

### 2.10 Histopathological examination

The left lung lobe was fixed in 10% formaldehyde. After fixation, the lung tissue was embedded in paraffin, sectioned to 5 μm thickness, and stained with hematoxylin-eosin stain (H&E). Then they were pathologically observed.

### 2.11 Measurement of inflammatory factors in serum and cell supernatant

The levels of IL-6, IL-1β and TNF-α in the serum and the cell supernatant were measured using the appropriate kits according to the manufacturer’s instructions.

### 2.12 Measurement of GSH, GPx, CAT, SOD, T-AOC, LDH, CRP, MPO, MDA and MIF in ALI Rats

The levels of GSH, GPx, CAT, SOD, T-AOC, LDH, CRP, MPO, MDA and MIF in the ALI rats were measured using the appropriate kits according to the manufacturer’s instructions.

### 2.13 Metabolomics analysis

#### 2.13.1 Rat serum sample preparation

Based on the above experimental results, control group, model group (LPS group), LPS + AEE high group (AEE, 216 mg·kg^−1^) were selected for metabolomic analysis. The serum was thawed at ambient temperature, acetonitrile (3:1, *V/V*) was added for precipitating the protein, then vortexed and mixed for 1 min and stood for 10 min. The samples were centrifuged at 14000 rpm for 10 min at 4°C. The supernatant was filtered through the 0.22 μm filter membrane and subsequently analyzed by UPLC-QTOF-MS/MS.

#### 2.13.2 UPLC-QTOF-MS/MS conditions

Liquid chromatography was performed on a 1290 UPLC system (Agilent Technologies Inc., California, USA). The LC conditions was as followed. The column was an Agilent ZORBAX SB C18 (2.1 × 150 mm, 1.8 μm) and the column temperature was 35°C. The injection volume was 2 μL and the autosampler temperature was set at 4°C. Mobile phase: 0.1% formic acid in water (A) and 0.1% formic acid in acetonitrile (B) at a flow rate of 0.3 mL·min^-1^. The gradient elution of A was as followed: 95% A from 0 to 3 min, 95 - 35% A from 3 to 4 min, 35 - 30% A from 4 to 7 min, 30 - 15% A from 7 to 10 min, 15 - 10% A from 10 to 15 min, 10 - 5% A from 15 to 18 min, and 18 to 20 min kept at 95% A.

Agilent 6530 Q-TOF (Agilent Technologies, USA) was used to carry out mass spectrometry of serum samples with a dual electrospray ionization source (ESI) operating in positive and negative ion modes. The fragment voltage was set to 135 V and the skimmer voltage was set to 65 V. In positive ionization mode, the capillary voltage was 4000 V while in negative ionization mode, it was 3500 V. The dry gas temperature was 350°C, and the flow rate was 10 L·min^-1^. The nebulizer pressure was set to 45 psi. The ions were scanned in the region of 50-1000 *m/z*.

#### 2.13.3 Metabolomics data analysis

The samples were tested by the UPLC/Q-TOF-MS system and the original data were obtained. The original data were processed using MS-DIAL V 4.38 to filter noise, baseline comparison, peak identification, data reduction, and normalization. Finally, the two-dimensional data matrix including mass-to-charge ratio, peak area and retention time were obtained. The obtained data were subjected to multivariate statistical analysis using SIMCA-P (version 13.0, Umetrics AB, Umea, Sweden) software, and the results were presented as principal component analysis (PCA) score plots and partial least squares discriminant analysis (OPLS-DA) score plots, respectively. The OPLS-DA model was also evaluated using the R^2^X, R^2^Y and Q^2^ parameters, and the permutation test was used to prevent overfitting of the model. R^2^Y can indicate the fit of the model and Q^2^ can indicate the predictability of the model, and the magnitude of these two parameters can reflect the reliability and accuracy of the model. Finally, the metabolites with VIP > 1 and *P* < 0.05 were screened for metabolite variability analysis in different groups. The screened differential metabolites were analyzed by targeted MS/MS using a UPLC/Q-TOF-MS analysis system, which combined their mass/charge (*m/z*) and retention time to obtain their accurate fragment ion information. Then, the information on the differential metabolites of concern was finalized by performing the spectral library comparison in METLIN and Human Metabolome Database (HMDB). According to the information on the differential metabolites, MetaboAnalyst 4.0 and KEGG database were used for the analysis of relevant metabolic pathways.

### 2.14 Statistical analysis

All data are presented as means ± SD. The differences among different treatment groups were analyzed with one-way ANOVA followed with Duncan’s multiple comparisons. Statistical significance was considered at *p*<0.05.

## 3 Results

### 3.1 Cytotoxicity of AEE and LPS

**Figure 1:**
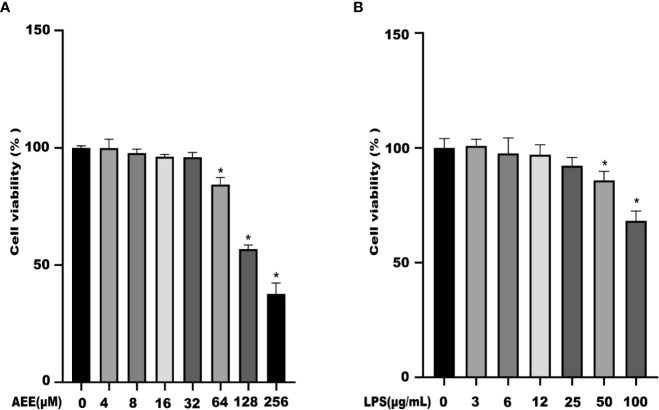
The effects of AEE and LPS with different concentrations on A549 cell viability, respectively. **(A)** AEE, **(B)** LPS. Values are presented as the means ± SD where applicable (*n* = 6). *P < 0.05 compared to control group.

The results showed that AEE (4 ~ 32 μM) had no cytotoxicity to A549 cells for 24 h. When the concentration was greater than 32 μM, the cell viability started to decrease (**Figure 1A**). Therefore, the concentrations of AEE below 32 μM were selected for subsequent experiments. It is known from **Figure 1B** that LPS had no cytotoxicity to A549 cells in the range of 3 to 12 μM for 24 h. When the concentration of LPS was 25 μM, the viability of A549 cells decreased slightly, therefore, the LPS concentration should be less than 25 μM for subsequent tests.

### 3.2 Effect of AEE on inflammatory cytokines in serum and cell supernatant

**Figure 2:**
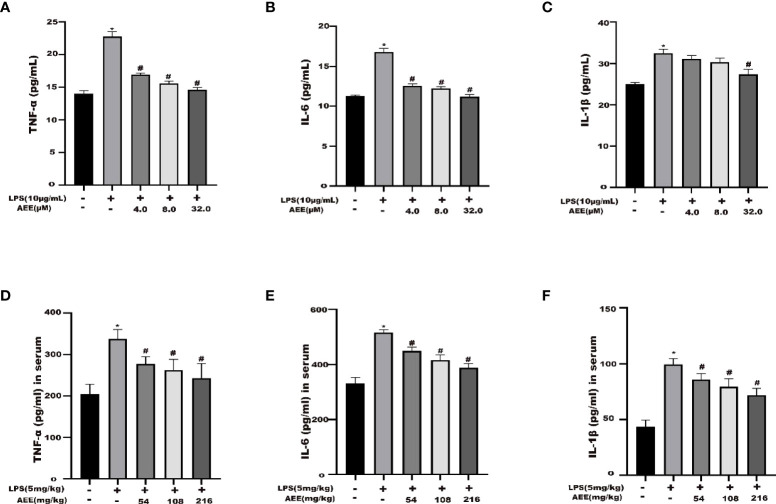
Effect of AEE on LPS-induced inflammatory cytokines. **(A)** TNF-α, **(B)** IL-6 and **(C)** IL-1β in the supernatant of A549 cells. **(D)** TNF-α, **(E)** IL-6, and **(F)** IL-1β in the serum of LPS-induced ALI rats. Values are presented as the means ± SD where applicable (*n* = 6). *P < 0.05 compared to control group, #*P* < 0.05 compared to LPS group.

The results (**Figure 2**) showed that the levels of IL-6, IL-1β, and TNF-α were higher in the LPS group than in the control group. The levels of IL-6, IL-1β, and TNF-α were decreased in the AEE-treated group in a dose-dependent manner compared with the LPS group. These data indicated that AEE could improve the inflammatory response to LPS *via* inhibiting the release of cytokines IL-6, IL-1β and TNF-α.

### 3.3 Effect of AEE on WBC count and total protein content in BALF

**Figure 3:**
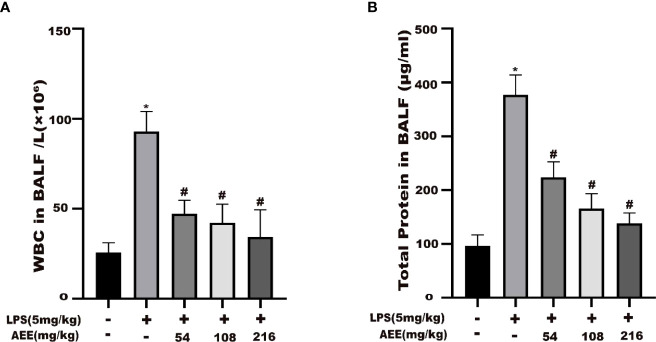
Effect of AEE on the number of WBC in BALF and total protein level in BALF of LPS-induced ALI rats. **(A)** WBC in BALF, **(B)** Total protein in BALF. Values are presented as the means ± SD where applicable (*n* = 8). *P < 0.05 compared to control group, #*P* < 0.05 compared to LPS group.

The results (**Figures 3A, B**) indicated that the WBC count and total protein content in the BALF of the LPS group were higher than those of the control group. Compared with the LPS group, the WBC count and total protein content in the BALF of the AEE-treated group were significantly decreased in the dose-dependence manner.

### 3.4 Effect of AEE on immune organ indices

**Figure 4:**
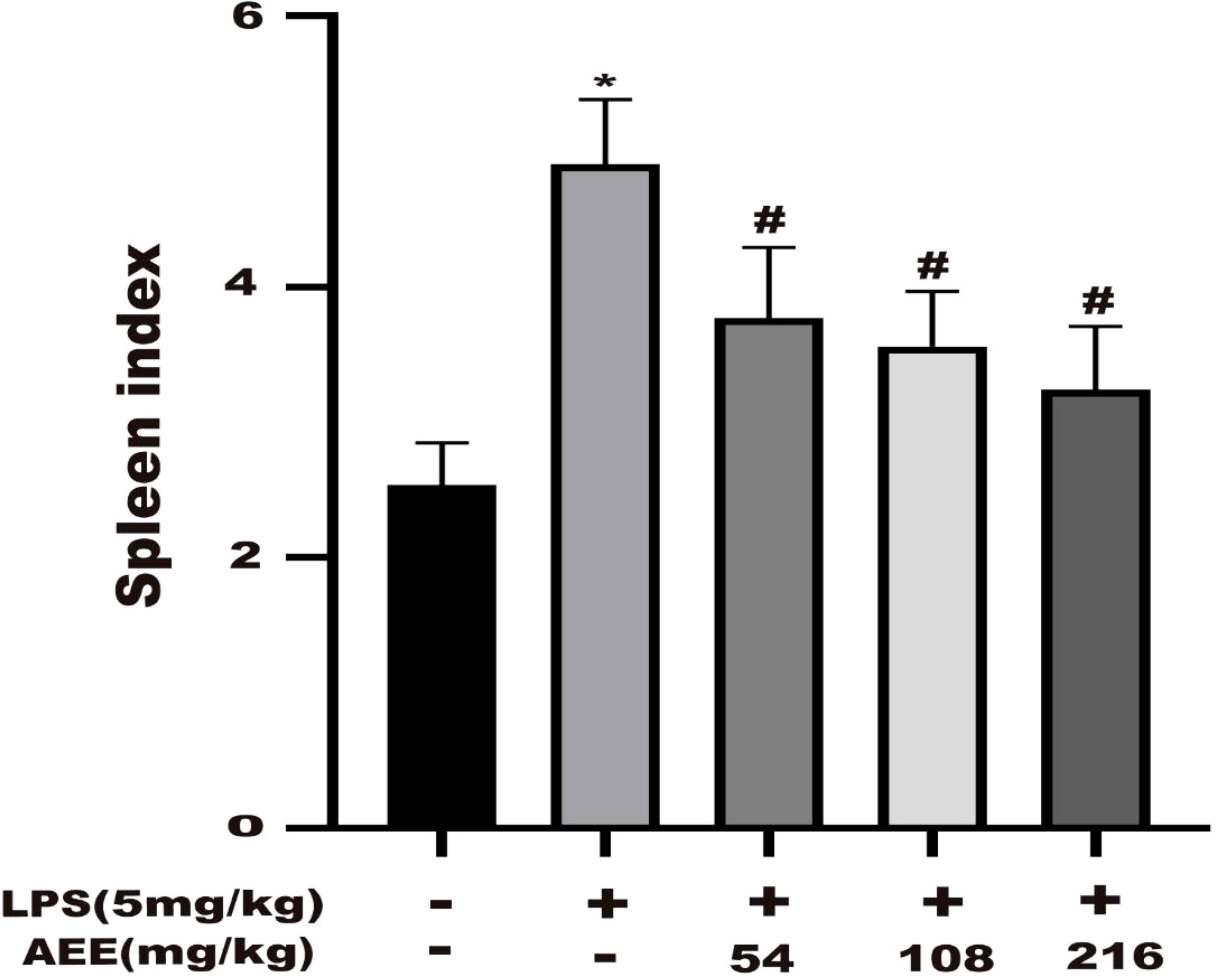
Effect of AEE on splenic index in LPS-induced ALI rats. Values are presented as the means ± SD where applicable (*n* = 8). *P < 0.05 compared to control group, #*P* < 0.05 compared to LPS group.

The effect of AEE on the splenic index of LPS-induced ALI in rats was investigated. The results (**Figure 4**) showed that the splenic index of the LPS group was higher than the control group. However, after AEE treatment, the spleen index of rats decreased significantly compared with the LPS group. Moreover, the decrease in spleen index was related to the dose of AEE.

### 3.5 Effect of AEE on lung W/D ratio

**Figure 5:**
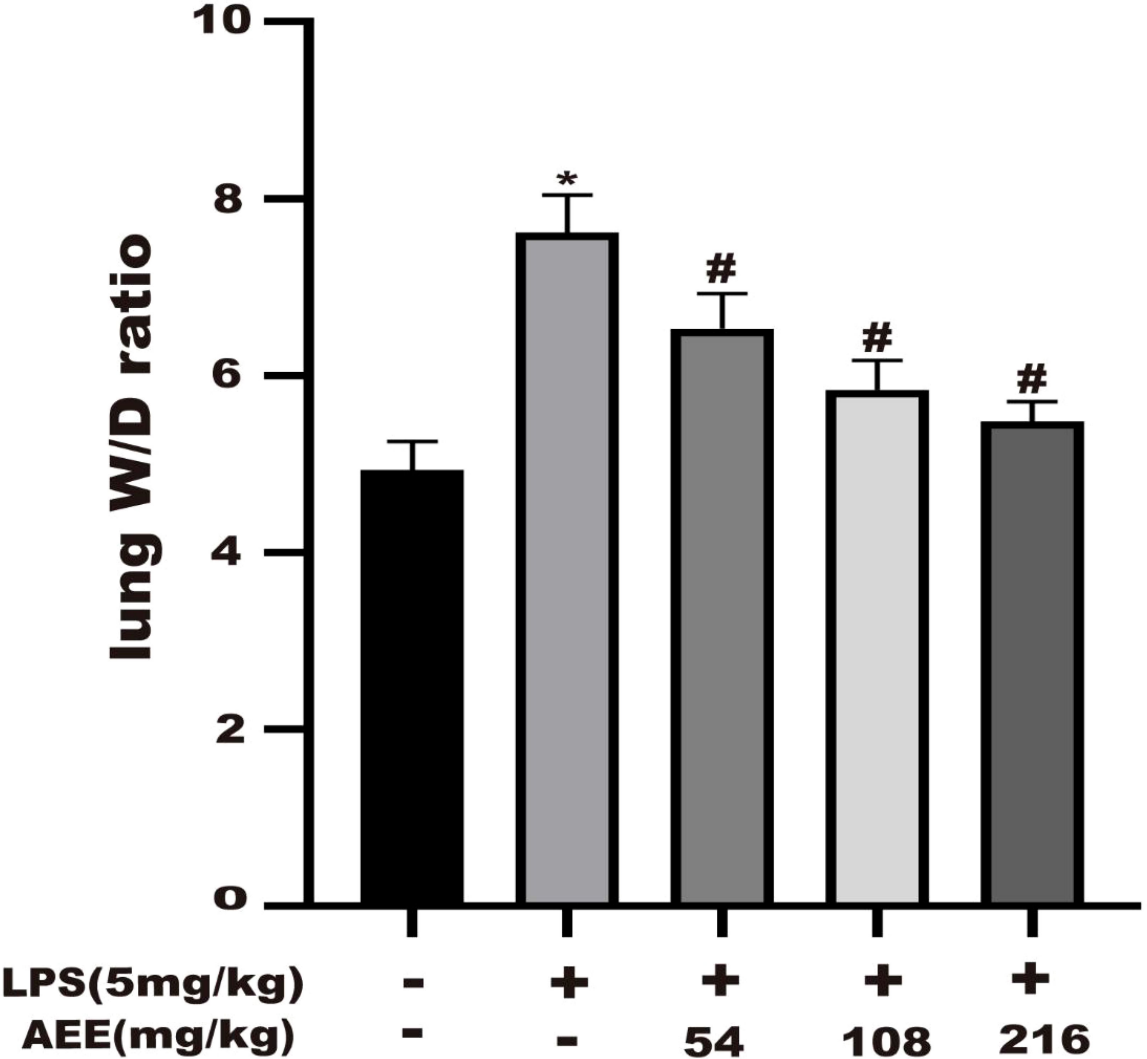
Effect of AEE on lung wet to dry weight ratio (W/D). Values are presented as the means ± SD where applicable (*n* = 8). *P < 0.05 compared to control group, #*P* < 0.05 compared to LPS group.

To evaluate the degree of edema in rat lungs, the wet to dry (W/D) weight ratio of lung tissue was calculated. As shown in **Figure 5**, compared with the control group, the W/D ratio of the LPS group was significantly increased, which indicated that LPS treatment significantly increased the W/D ratio. However, the W/D ratio of lung tissue was significantly decreased in a dose-dependent manner after AEE treatment compared with the LPS group.

### 3.6 Effects of AEE on the levels of CRP, MPO and MIF in ALI rats

**Figure 6:**
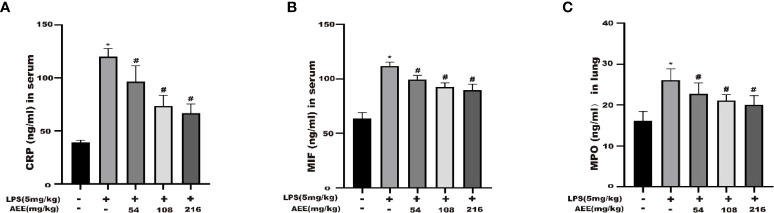
Effect of AEE on concentrations of CRP **(A)**, MIF **(B)** in serum, MPO **(C)** in lung tissue. Values are presented as the means ± SD where applicable (*n* = 8). *P < 0.05 compared to control group, #*P* < 0.05 compared to LPS group.

The results (**Figures 6A–C**) showed that the levels of CRP, MPO and MIF in the serum of rats in the LPS group were higher than in the control group. Compared with the LPS group, there were significant decreases in the levels of CRP, MPO, and MIF in the serum of rats in the AEE-treated group. All these data indicated that AEE could prevent lung inflammation during ALI in rats by decreasing CRP, MPO, and MIF in a dose-dependent manner.

### 3.7 Effect of AEE on oxidative stress markers in LPS-induced ALI rats

**Figure 7:**
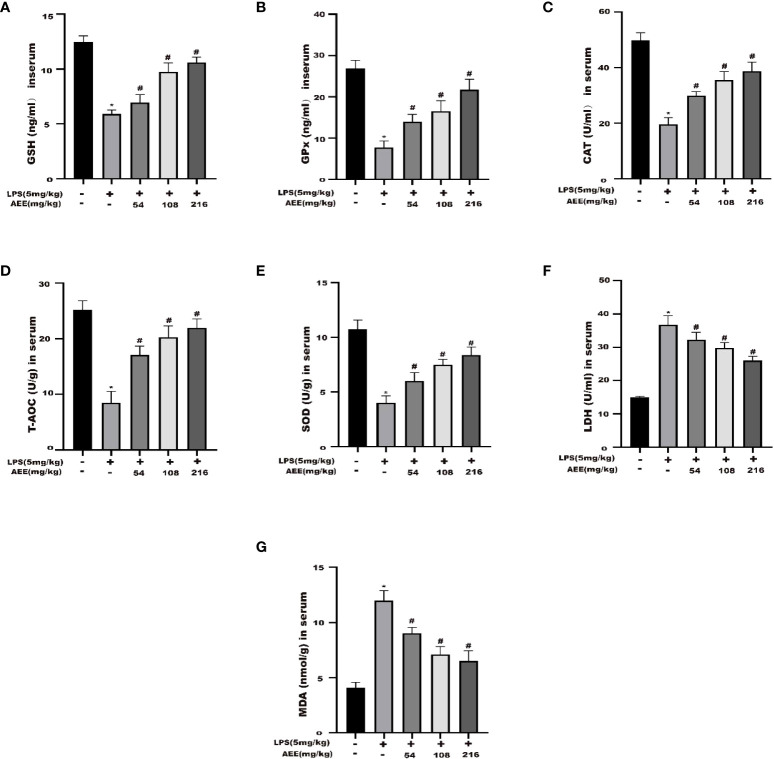
Effect of AEE on the level of oxidative stress in LPS-induced ALI rats. **(A)** GSH, **(B)** GPx, **(C)** CAT, **(D)** T-AOC, **(E)** SOD, **(F)** LDH, **(G)** MDA. Values are presented as the means ± SD where applicable (*n* = 8). *P < 0.05 compared to control group, #*P* < 0.05 compared to LPS group.

To explore the anti-oxidative stress effect of AEE on LPS-induced ALI in rats, the following oxidative stress indicators were selected: GSH, GPx, CAT, T-AOC, SOD, LDH, and MDA. As shown in **Figures 7A–G**, compared with the control group, GSH, GPx, CAT, T-AOC, and SOD levels were significantly decreased in the LPS group, while LDH and MDA levels were significantly increased. Compared with the LPS group, the levels of GSH, GPx, CAT, T-AOC, and SOD in the AEE-treated group increased significantly in a dose-dependent manner, while the levels of LDH and MDA decreased significantly.

### 3.8 Analysis of histopathological changes in the lung

**Figure 8:**
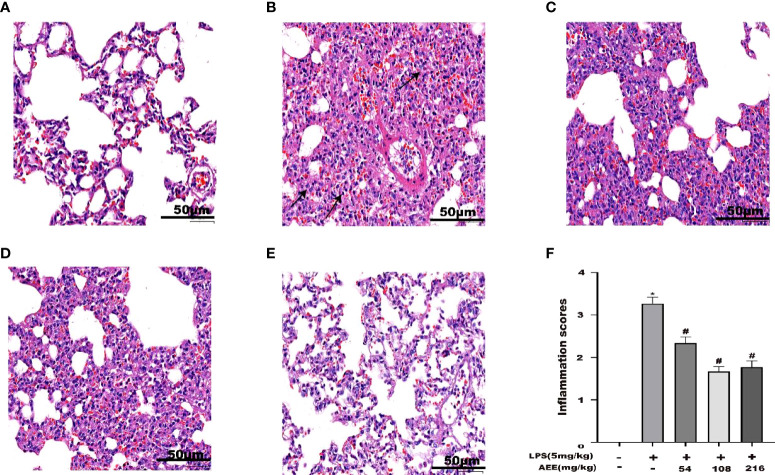
Effect of AEE on histopathological changes in the lungs of LPS-induced ALI rats. **(A)** Control group, **(B)** LPS group, **(C)** LPS + AEE group (AEE, 54 mg kg^-1^), **(D)** LPS + AEE group (AEE, 108 mg kg^-1^), **(E)** LPS + AEE group (AEE, 216 mg kg^-1^), **(F)** Inflammatory change score chart. *P < 0.05 compared to control group, ^#^ *P* < 0.05 compared to LPS group.

After HE staining of lung tissue, histopathology was observed. As shown in **Figure 8**, compared with the control, the LPS group showed necrosis of alveolar epithelial cells, the disintegration of nuclei, proliferation of alveolar epithelial cells, and infiltration of inflammatory cells, mainly neutrophils, which were seen in the necrotic area and in the interstitial space. Compared with the LPS group, AEE-treated groups all had some degree of reducing LPS-induced lung histopathological damage in a dose-dependent manner.

### 3.9 Metabolomic analysis of the effect of AEE on LPS-induced ALI in rats

#### 3.9.1 Analysis of serum metabolites

**Figure 9:**
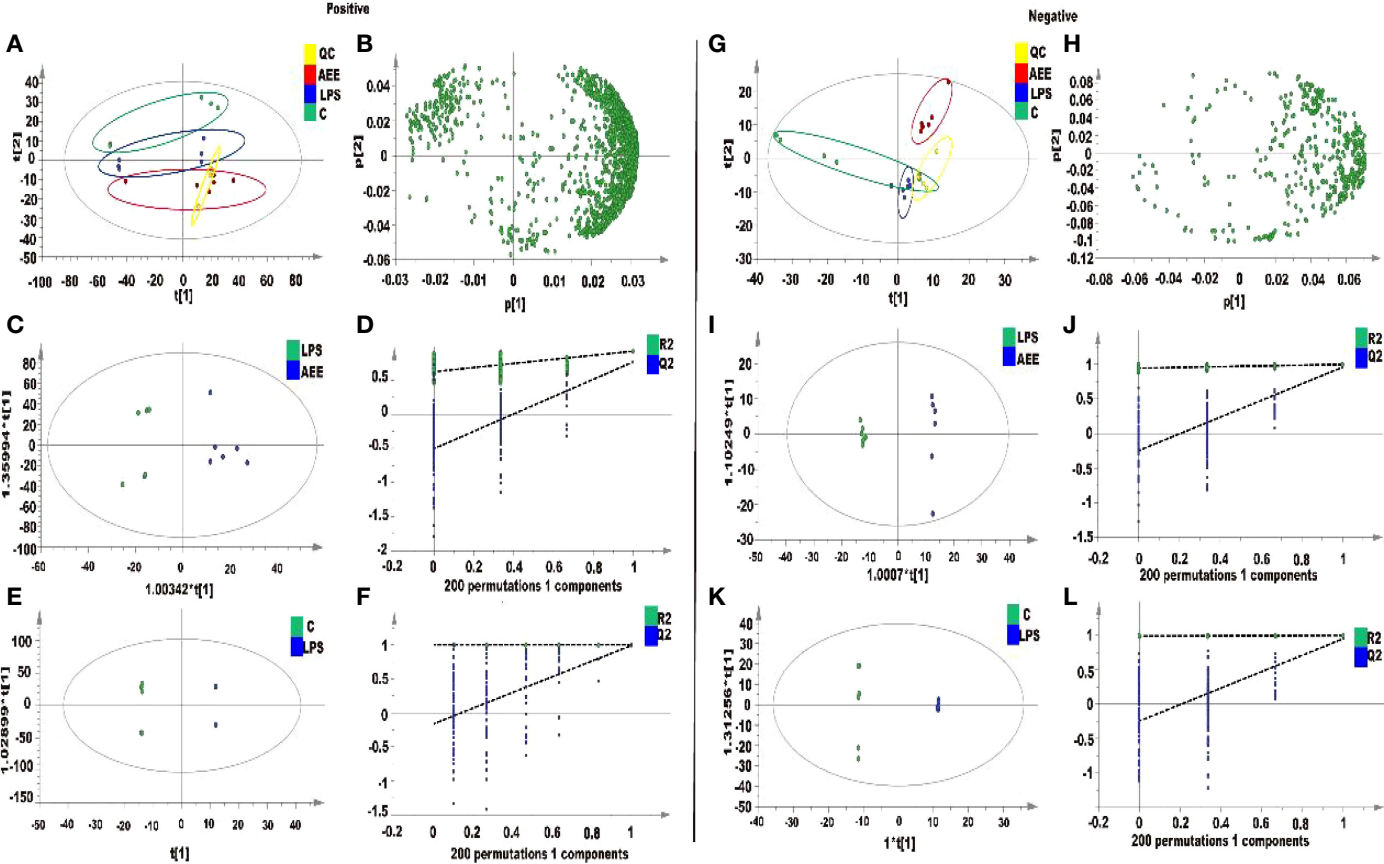
Metabolomic analysis of the effect of AEE on LPS-induced ALI in rats. **(A, G)** PCA score plots based on rat sera obtained from control, LPS and AEE groups in positive and negative modes, ESI+: R^2^ = 0.878, ESI-: R^2^ = 0.829. **(B, H)** The loading plots of AEE and LPS groups in positive and negative modes. **(C, I)** OPLS-DA score plots for AEE and LPS groups in positive and negative models, ESI+: R^2^ X = 0.807, R^2^ Y = 0.926, Q^2^ = 0.768; ESI-: R^2^ X = 0.646, R^2^ Y = 0.998, Q^2^ = 0.958. **(D, J)** Permutation test for the OPLS-DA model, ESI+: intercepts of R^2^ = 0.626 and Q^2^ = -0.502 and ESI-: intercepts of R^2^ = 0.946 and Q^2^ = -0.245. **(E, K)** OPLS-DA score plots for control and LPS groups in positive and negative modes, ESI+: R^2^ X = 0.977, R^2^ Y = 1, Q^2^ = 0.992; ESI-: R^2^ X = 0.887, R^2^ Y = 1, Q^2^ = 0.951. **(F, L)** Permutation test for the OPLS-DA model, ESI+: intercepts of R^2^ = 0.999 and Q^2^ = -0.16, ESI-: intercepts of R^2^ = 0.991 and Q^2^ = -0.243. Values are presented as the means ± SD where applicable (*n* = 6).

Metabolite data of serum samples obtained from three experimental groups were further analyzed by principal component analysis (PCA). As shown in **Figures 9A, B, G, H**, there were significant changes in the metabolite profiles of the serum samples after drug administration. Under the positive and negative modes, the first two principal components explained 87.8% and 82.9% of the total variance, respectively, which showed a significant separation. To further decrease the effect of irrelevant factors on the analysis results and improve the separation and identification of metabolites, the effects of AEE on metabolites in LPS-induced AIL rats were investigated, and three experimental groups were modeled and analyzed using orthogonal partial least squares discriminant analysis (OPLS-DA). Moreover, the OPLS-DA models of each group were validated with the permutation tests. The parameters of the permutation tests for the OPLS-DA model for each group were shown in **Figures 9C, E, I, K**. The OPLS-DA models were analyzed between the LPS group and the other groups. Results of the OPLS-DA score plot showed a clear separation between the LPS group and the other groups and there were no overlaps in either the positive or negative mode. the R^2^X, R^2^Y and Q^2^ values of the OPLS-DA model indicated that the model was not overfitted, which matched the sample reality and had predictive power (**Figures 9D, F, J, L**).

The potential differential metabolites were selected by VIP (VIP > l) and t-test *P* < 0.05 in the OPLS-DA model, and the differential metabolites were analyzed by Target MS/MS scan. To obtain secondary characteristic ion fragments of the differential metabolites for comparison in relevant databases. As shown in **Table 1**, 18 differential metabolites were finally screened in the serum sample analysis, including 2-Methylglutaric acid, Suberylglycine, Spermidine, Doxylamine, Delphinidin, D-Alanine, Gamma-glutamylcysteine, 2’-Deoxyguanosine-5’-monophosphate, Pterin, N-Acetyl-L-tyrosine, Benzyl benzoate, Flavone, L-Histidinol, Mannitol 1-phosphate, m-Coumaric acid, 5-Aminopentanoic acid, Benzocaine, Ferulic acid. After AEE treatment, the levels of these differential metabolites were normalized due to up- or down-regulation, which indicated that AEE could improve the metabolic bias caused by LPS in rats.

**Table 1:** Statistics of differential metabolites in serum of rats.

No	RT	VIP	Formula	Metabolites	SM	m/z	Fold Change	
							C/LPS	AEE/LPS
1	1.005	1.59012	C_6_H_10_O_4_	2-Methylglutaric acid	ESI+	172.9855	0.6121	1.2362^*^
2	1.038	1.94976	C_10_H_17_NO_5_	Suberylglycine	ESI+	231.0259	0.488312	1.98684^*^
3	1.543	1.39598	C_3_H_7_NO_2_	D-Alanine	ESI+	89.0691	0.498363	1.92213^*^
4	1.754	1.21166	C_11_H_13_NO_4_	N-Acetyl-L-tyrosine	ESI+	223.0004	0.67483	1.48305^*^
5	2.798	1.39284	C_8_H_14_N_2_O_5_S	Gamma-glutamylcysteine	ESI+	250.0265	0.8496	2.93015^*^
6	2.858	1.25671	C_10_H_14_N_5_O_7_P	2’-Deoxyguanosine 5’-monophosphate	ESI+	347.1064	2.73388	0.691961^*^
7	4.371	1.50733	C_7_H_19_N_3_	Spermidine	ESI+	145.1194	0.237651	0.906861^*^
8	7.926	1.24692	C_15_H_11_O_7_	Delphinidin	ESI+	303.0553	0.749837	1.97929^*^
9	9.071	1.31621	C_6_H_5_N_5_O	Pterin	ESI+	163.0342	0.724711	2.00309^*^
10	9.079	1.61923	C_14_H_12_O_2_	Benzyl benzoate	ESI+	212.0539	1.70343	1.85006
11	11.896	2.00916	C_17_H_22_N_2_O	Doxylamine	ESI+	270.2728	2.80768	1.4496^*^
12	19.496	1.57759	C_6_H_13_Cl_2_N_3_O	L-Histidinol	ESI+	141.1103	0.608435	0.98478^*^
13	1.687	1.13524	C_6_H_15_O_9_P	Mannitol 1-phosphate	ESI-	262.0628	1.88564	2.8718^*^
14	4.552	1.02446	C_15_H_10_O_2_	Flavone	ESI-	222.0314	0.830361	1.55238^*^
15	4.645	1.09507	C_9_H_8_O_3_	m-Coumaric acid	ESI-	164.0726	1.48184	0.635764^*^
16	5.563	1.20957	C_5_H_9_NO_3_	5-Aminopentanoic acid	ESI-	117.054	1.81853	2.71211^*^
17	6.391	1.06687	C_9_H_11_NO_2_	Benzocaine	ESI-	165.0172	0.47309	1.29626^*^
18	12.136	1.30167	C_10_H_10_O_4_	Ferulic acid	ESI-	194.0847	0.789345	1.50953^*^

RT, retention time; VIP, variable importance in the projection; SM, scan mode; +, metabolites identified in positive mode; -, metabolites identified in negative mode. Metabolites identified in both positive and negative modes; *P < 0.05 compared to LPS group; C/LPS, control group compared with LPS group; AEE/LPS, AEE group compared with LPS group.

#### 3.9.2 Metabolic pathway analysis

**Figure 10:**
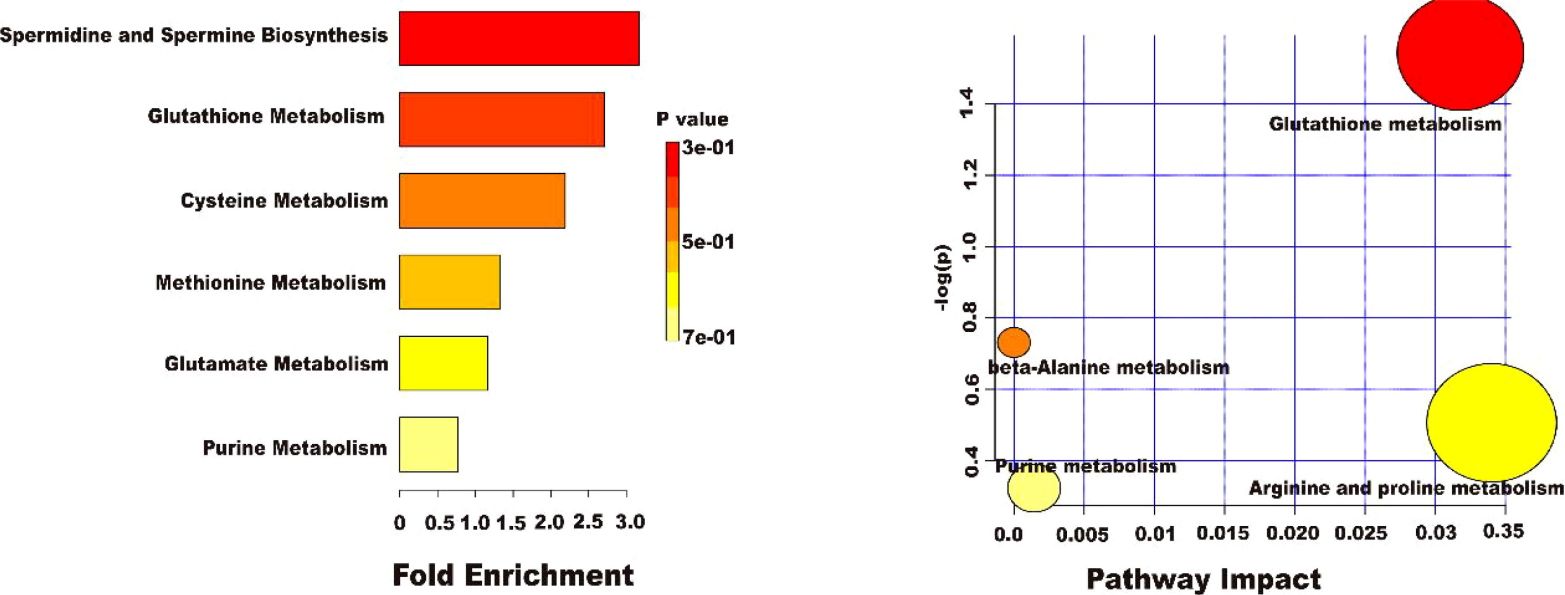
Results of ploidy enrichment and pathway analysis of potential metabolites in serum.

To more deeply investigate the effect of AEE on the metabolic pathways in the organism of LPS-induced ALI rats, the metabolomic pathways were analyzed using MetaboAnalyst 5.0. **Figure 10** showed an overview of the metabolic pathway analysis in the form of bar and bubble charts. There were 6 main metabolic pathways: Spermidine and Spermine Biosynthesis, Glutathione Metabolism, Cysteine Metabolism, Methionine Metabolism, Glutamate Metabolism, and Purine Metabolism. The effects of pathways were mainly focused on Glutathione metabolism, beta-Alanine metabolism, Arginine and proline metabolism, and Purine metabolism. The LPS-induced ALI in rats was mainly reflected in oxidative stress and energy metabolism. The results indicated that LPS-induced ALI caused metabolic disorders and oxidative stress in rats and AEE could alleviate it to some extent.

## 4. Discussion

With the development of society, the problem of environmental pollution is becoming more and more serious, especially air pollution (36). According to the statistics of relevant organizations, the number of deaths caused by air pollution is up to 7 million per year worldwide (37). The fine particles in the air and other harmful substances can enter the organism with breathing, which leads to the activation of the body’s immune system and further induces oxidative stress, inflammatory reactions, and local tissue damage (38). This increases the occurrence of various respiratory diseases, including asthma, respiratory tract infections, decreased lung function, and acute lung injury, which seriously affects people’s health (39–43). Therefore, this experiment investigated the protective effect of AEE on LPS-induced inflammation in A549 cells and the protective effect of AEE on LPS-induced ALI in rats. The effects of AEE on ALI were investigated from both *in vitro* assays and *in vivo* experiments to provide the theoretical basis for the comprehensive and rational use of AEE.

In this study, the LPS concentration of 10 μg·mL^-1^ was chosen to induce the inflammation model in A549 cells based on the results of the cytotoxicity assay. Previous studies also showed that LPS worked at a concentration of 10 μg·mL^-1^ on A549 cells (44), and the results of this assay were also consistent with them. According to the toxicity of AEE on A549 cells, three concentrations of the low, medium, and high (4,8,32 μM) were chosen for the follow-up experiments. IL-6, IL-1β, and TNF-α act as pro-inflammatory factors, which are involved in the inflammatory response when the cells are attacked by LPS (45). Compared with the control, the levels of IL-6, IL-1β, and TNF-α in the supernatant of cells in the LPS group were significantly increased, indicating that A549 cells occurred an inflammatory response. Compared with the LPS group, the levels of IL-6, IL-1β and TNF-α were significantly decreased in the AEE-treated group, which showed a dose-dependent effect. This indicated that AEE had a protective effect on inflammation in A549 cells.

According to the preliminary results of *in vitro* experiments, *in vivo* experiments were also conducted. Experimental animals were selected as Wistar rats, and the modeling drug was chosen as LPS with the concentration of 5 mg·kg^-1^ (46, 47).

The results in this study showed that AEE could reduce LPS-induced lung inflammation and injury in rats. Compared with the LPS group, AEE significantly decreased the serum levels of IL-6, IL-1β, and TNF-α, which indicated that AEE could reduce the release of inflammatory mediators in the blood to some extent and alleviate the damage to lung tissue caused by LPS.

C-reactive protein (CRP) is a protein that rises sharply in response to infection or tissue damage in the body (48). In this study, the serum CRP level was higher in the LPS group than the control group, indicating the presence of acute inflammation in the rats. Compared with the LPS group, the AEE group showed a significant decrease in CRP content, which indicated the therapeutic effect of AEE on acute inflammation. Myeloperoxidase (MPO) is a hemoglobin peroxidase expressed most richly in neutrophils (49, 50). As essential mediators of the innate immune system, neutrophils were rapidly recruited to sites of inflammation, and recognized, phagocytosed, inactivated foreign microorganisms, playing a pivotal role in host defense (51, 52). The level of MPO was positively correlated with the number of neutrophils (53). During infection, MPO is involved in the inflammatory response, but it can cause oxidative reactions and the formation of large amounts of oxides, which may cause damage to the tissues (54). The MPO level in the LPS group was higher than in the control group. However, the level of MPO in the AEE group was lower than in the LPS group, which suggested that AEE could reduce the harmful effects of inflammation on the lungs. Neutrophil aggregation is a key character of inflammation in the lung. MPO is a marker of neutrophil function and activation and its activity level can implicitly reflect the extent of neutrophil aggregation at the site of inflammation (46). Therefore, neutrophils were not tested in this study, but the changes in neutrophils could be inferred from the changes in MPO. Macrophage migration inhibitory factor (MIF) is a pro-inflammatory cytokine that is expressed in a variety of cells (55). MIF plays a pathogenic role in many inflammatory diseases, including acute and chronic inflammation, sepsis (56). AEE reduced MIF levels, which further confirmed the protective effect of AEE against inflammation. Certainly, if a number of macrophages and neutrophils were tested and analyzed with the levels of MPO and MIF, the results were more definite and perfect.

One of the main characteristics of ALI is pulmonary edema (57). The primary cause of pulmonary edema is endothelial damage due to inflammatory response, resulting in microvascular leakage (58, 59). The lung W/D ratio is an important indicator of pulmonary edema (60). The increase in BALF protein concentration due to protein extravasation is another important indicator of pulmonary edema (61). Therefore, to evaluate the therapeutic effect of AEE on pulmonary edema, the following experiments were conducted. The result showed that AEE group significantly decreased the lung W/D ratio compared with the LPS group, which indicated that AEE inhibited the accumulation of fluid in the lung tissue. The detection of protein concentration in BALF showed that AEE group significantly reduced the protein concentration in BALF, which indicated that AEE inhibited protein extravasation in lung tissue. Assaying the WBC count in BALF showed that the WBC count in BALF decreased in AEE group, which further suggested a therapeutic effect of AEE on inflammation. In addition, morphological changes in lung tissue were observed by H&E staining. The results showed that AEE group alleviated the pathological changes such as alveolar epithelial cell proliferation, inflammatory cell infiltration, and alveolar epithelial cell necrosis to some extent.

The non-normal immune response of the organism is an essential factor in triggering ALI (62). The spleen is the most important immune organ of the body, and its relative proportion is an essential indicator of nonspecific immunity, they play an active role in non-specific immunity in immune defense (63, 64). As the results showed, compared with the LPS group, the spleen index of rats in AEE group was significantly lower, which indicated that AEE had a protective effect on the spleen.

According to studies, the inflammatory response may trigger oxidative stress, and oxidative stress can exacerbate the inflammatory response, which can interact with each other in multiple ways (65–67). Several studies showed that oxidative stress and the inflammatory response were essential factors in triggering lung injury (68, 69). MDA is a product of lipid oxidation under oxidative stress conditions, which has a damaging effect on cells (70). SOD is the most important free radical scavenger in the body, maintaining the metabolic homeostasis of the body (71). LDH is a crucial oxidoreductase in organisms that is an indicator of oxidative stress. CAT and GSH are essential non-protein antioxidants that clears lipid peroxide radicals. GPx clear oxidation products and is associated with the transport of GSH. T-AOC is the total antioxidant level consisting of various antioxidant substances and antioxidant enzymes (72–74). Some results showed that in LPS-induced ALI, MDA levels increased while antioxidant activity decreased (19, 75, 76). In the study, the above indices were chosen to evaluate the protective effect of AEE on oxidative stress in rats. Compared with the LPS group, the levels of MDA and LDH were significantly decreased and the levels of SOD, CAT, GSH, GPx, and T-AOC were somewhat restored in the AEE group. It indicated that the protective effect of AEE on LPS-induced ALI in rats might be its antioxidant capacity.

The action mechanism of AEE on the serum of ALI rats were investigated by an untargeted metabolomics approach using UPLC-Q-TOF/MS. The results showed that the mechanism of AEE on ALI may mainly involve Spermidine and Spermine Biosynthesis, Glutathione Metabolism, Cysteine Metabolism, Methionine Metabolism, Glutamate Metabolism, and Purine Metabolism. Spermidine and spermine biosynthesis may be one of the metabolic pathways through which AEE plays a protective role in LPS-induced ALI. Spermidine and spermine can alleviate oxidative stress, and spermidine is involved in protecting cells from oxidative stress by clearing ROS (77–79). The biosynthetic pathway of spermidine was inhibited in the LPS group, while the production of spermidine was increased in the AEE group, which suggested that AEE might alleviate LPS-induced ALI in rats by clearing excess ROS. Cysteine metabolism, methionine metabolism, and glutathione metabolic pathways are closely related to oxidative stress (80). Methionine residues can also act as endogenous antioxidants, which can be used as a marker that the body is in a state of oxidative stress (81). Glutathione (γ-glutamyl-cysteinyl-glycine, GSH) plays an important role in the antioxidant process because of its ability to effectively clean up free radicals and other reactive oxygen species (82). The changes in GSH levels indicate changes in the oxidative status of the body, and when the body is stimulated by oxidation, GSH synthesis increases (83). The metabolomic results showed that the biosynthetic pathway of Gamma-glutamylcysteine was inhibited in the LPS group, while the production of Gamma-glutamylcysteine was increased in the AEE group. Purine metabolism is an important response to oxidative stress, and abnormal purine metabolism can generate damaging reactive oxygen species (ROS) (84, 85). 2’-Deoxyguanosine 5’-monophosphate production was increased in the LPS group, indicating oxidative stress in rats. In contrast, 2’-Deoxyguanosine 5’-monophosphate production was decreased in the AEE group, which suggested an antioxidant effect of AEE. In the subsequent study, we will further explore the action mechanism of AEE on ALI in rats and elucidate the signaling pathway of AEE action on ALI in rats.

## 5. Conclusion

AEE had a protective effect against LPS-induced ALI in rats. *In vitro* and *in vivo* experiments demonstrated that AEE can reduce inflammation in rat lung by decreasing the release of pro-inflammatory factors as IL-6, IL-1β, and TNF-α. AEE alleviated pulmonary edema in rats by inhibiting lung tissue protein exudation, decreasing lung wet-to-dry (W/D) weight ratio, decreasing immune organ index, and improving lung histopathology. In addition, AEE showed an alleviating effect on inflammation-induced oxidative stress, which suggested an anti-oxidative stress effect of AEE. From metabolomics, AEE potential mechanism was related to the regulation of oxidative stress and metabolic pathway disorders of energy metabolism. Therefore, AEE could be used as a potential drug to improve ALI.

## Data availability statement

The original contributions presented in the study are included in the article/supplementary material. Further inquiries can be directed to the corresponding authors.

## Ethics statement

The animal study was reviewed and approved by Institutional Animal Care and Use Committee of Lanzhou Institute of Husbandry and Pharmaceutical Science of Chinese Academy of AgriculturalSciences.

## Author contributions

J-YL and Y-JY designed the experiments and revised the manuscript. QT and Z-DZ designed and performed the experiments, and wrote the manuscript. X-WL synthesized and purified AEE. L-XB, S-HL, W-BG and ZQ supplied reagents. All authors contributed to the article and approved the submitted version.

## Funding

This study was supported by the Special Fund for National natural science foundation of China (Grant/Award Numbers: 31872518).

## Conflict of interest

The authors declare that the research was conducted in the absence of any commercial or financial relationships that could be construed as a potential conflict of interest.

## Publisher’s note

All claims expressed in this article are solely those of the authors and do not necessarily represent those of their affiliated organizations, or those of the publisher, the editors and the reviewers. Any product that may be evaluated in this article, or claim that may be made by its manufacturer, is not guaranteed or endorsed by the publisher.

## Abbreviations

AEE, Aspirin eugenol ester; LPS, Lipopolysaccharide; ALI, Acute lung injury; TNF-α, Tumor necrosis factor-α; IL-6, Interleukin-6; IL-1β, Interleukin-1β; WBC, White blood cell; W/D, Lung wet-to-dry weight; BALF, Bronchoalveolar lavage fluid; HE, Hematoxylin-Eosin; FBS, fetal bovine serum; NEAA, Non-essential amino acids; CCK-8, Cell counting kit-8; BCA, Bicinchoninic acid; GSH, Glutathione; GPx, Glutathione peroxidase; CAT, Catalase; SOD, Superoxide dismutase; T-AOC, Total antioxidant capacity; LDH, Lactate dehydrogenase; CRP, C-reactive protein; MPO, Myeloperoxidase; MDA, Malondialdehyde; MIF, Macrophage mobility inhibitory factor; CMC-Na, Carboxymethylcellulose.
